# Learning From Human Responses to Deconditioning Environments: Improved Understanding of the “Use It or Lose It” Principle

**DOI:** 10.3389/fspor.2021.685845

**Published:** 2021-12-03

**Authors:** David A. Hart

**Affiliations:** ^1^Bone and Joint Health Strategic Clinical Network, Alberta Health Services, Edmonton, AB, Canada; ^2^Department of Surgery, Faculty of Kinesiology, University of Calgary, Calgary, AB, Canada; ^3^McCaig Institute for Bone and Joint Health, University of Calgary, Calgary, AB, Canada; ^4^Department of Family Practice, Centre for Hip Health and Mobility, University of British Columbia, Vancouver, BC, Canada

**Keywords:** exercise and deconditioning, menopause, prolonged bedrest, obesity, space flight, ground reaction forces

## Abstract

Physical activity, mobility or patterned mobility (i.e., exercise) is intrinsic to the functioning of *Homo sapiens*, and required for maintenance of health. Thus, systems such as the musculoskeletal and cardiovascular systems appear to require constant reinforcement or conditioning to maintain integrity. Loss of conditioning or development of chronic deconditioning can have multiple consequences. The study of different types of deconditioning and their prevention or reversal can offer a number of clues to the regulation of these systems and point to how deconditioning poses risk for disease development and progression. From the study of deconditioning associated with spaceflight, a condition not predicted by evolution, prolonged bedrest, protracted sedentary behavior, as well as menopause and obesity and their consequences, provide a background to better understand human heterogeneity and how physical fitness may impact the risks for chronic conditions subsequent to the deconditioning. The effectiveness of optimized physical activity and exercise protocols likely depend on the nature of the deconditioning, the sex and genetics of the individual, whether one is addressing prevention of deconditioning-associated disease or disease-associated progression, and whether it is focused on acute or chronic deconditioning associated with different forms of deconditioning. While considerable research effort has gone into preventing deconditioning, the study of the process of deconditioning and its endpoints can provide clues to the regulation of the affected systems and their contributions to human heterogeneity that have been framed by the boundary conditions of Earth during evolution and the “use it or lose it” principle of regulation. Such information regarding heterogeneity that is elaborated by the study of deconditioning environments could enhance the effectiveness of individualized interventions to prevent deconditions or rescue those who have become deconditioned.

## Introduction

All humans function within a “physiological window” of physical activity that is defined at the individual level for a number of physiological systems, including the musculoskeletal system (reviewed in Turner, 1991). However, heterogeneity of the population means that some can function better in specific activities (e.g., elite athletics) and endeavors than others. It is also well-known that if one exceeds this individualized physiological window of function, it can lead to injury in the acute situation, but also modification of systems if they are chronically over-used during activities (discussed in Thornton and Hart, 2011). Conversely, acute and chronic under-use of many biological systems such as those of the musculoskeletal and cardiovascular systems can lead to atrophy and dysfunction. For example, removal of a knee meniscus from the *in vivo* loading environment leads to the rapid de-repression of a set of catabolic genes (Natsu-ume et al., 2005). Thus, all humans require repeated physical activity within a physiological window to function properly across the lifespan and this can be defined as continual conditioning of the integrated systems. Conversely, the under-utilization of systems can lead to alterations and their consequences in disease development and can be termed “deconditioning.”

For the sake of this review, the term “deconditioning” refers to circumstances where the amount of mechanical loading of various biological systems falls below a threshold required to maintain system integrity as defined by evolutionary requirements for a complex system operating in a 1 g environment. “Conditioning” refers to the amount of physical activity or patterned physical activity (e.g., exercise) required to maintain biological systems operating in a 1 g environment across the lifespan.

While it is clear that chronic deconditioning of essential biological systems should be avoided to minimize risk for development of disease, it is also clear that studying how deconditioning that affects essential systems can provide clues to the regulation of those systems by mechanical and biological variables that may otherwise not be readily elucidated. Therefore, the premise of this review is to discuss some of the various forms of deconditioning environments, and how they can be used to assess the involvement of human heterogeneity in the biological responses to such environments in relation to systems that evolved under the boundary conditions of Earth. Differences between acute and chronic deconditioning and their potential reversibility is also discussed as there are likely important distinctions between the two scenarios regarding their implications for health.

## The Need For Constant Reinforcement of Biological Conditioning Is Embedded In Human Evolutionary History

While it may be intuitive to some, many people appear to overlook the fact that the *Homo sapiens* of today are the product of millions of years of evolution, with the past ~100,000 years critical to the current version of “humans.” Thus, we are the result of the retention of ~20,000 genes and associated mutations that have contributed to our survival in general or in specific environments, as well as a myriad of regulatory elements which distinguish humans from their evolutionary relatives, all in the context of the boundary conditions of Earth. Key features of those genetic contributions relate to mechanical functioning via walking upright, exhibiting mobility/locomotion and patterned mobility (navigation through the environment), and an ability to adapt to a changing environment. A key feature of humans is that they are very heterogeneous genetically, but have retained their commonalities to assure their survival under different conditions through evolution.

Of those features on the above list, likely walking upright, and developing effective mobility and coordinated eye-leg movement to navigate the environment were critical for successful hunting, avoiding predators, and nomadic lifestyles to minimize issues surrounding food security or adverse environmental conditions. Walking upright needed adaptations to both the vascular and cardiac systems, as well as the neuro-control systems required for patterned mobility and regulation of the cardiovascular system, the respiratory system, and metabolic systems. Thus, all of the genes currently contributing to the functioning of *Homo sapiens* were developed and refined in the context of the boundary conditions of the Earth, including a 1 g gravitational field, a somewhat dynamic geomagnetic field, a variable location-specific background radiation, and a viable temperature spectrum, to name a few. Therefore, these systems had to either evolve to neutralize the influence of the background boundary conditions, or incorporate them into their control mechanisms. Furthermore, as many systems depend in part on mobility to maintain their integrity, systems such as the cardiovascular systems, respiratory, musculoskeletal system, and the brain, sustaining these systems via physical activity, exercise and mobility in the context of a 1 g environment is a central tenet (reviewed in Hart, 2018a; Hart and Zernicke, 2020).

Likely, much of the variation in genes essential for functioning within the boundary conditions of Earth are silent as long as the humans operate within those conditions. It is also clear that continued use of these evolutionarily defined attributes for system regulation likely require continued reinforcing or conditioning of the physiological systems. As will be discussed below, many, if not all of the systems mentioned subscribe to the “use it or lose it” paradigm, and thus continual conditioning of the systems via use/conditioning (during evolution) or exercise (modern societies) of these physiological systems is required to maintain system integrity. Support for this premise has been garnered from the study of environments where “deconditioning” occurs and has detrimental impact on human health, including that of the cardiovascular system.

“Deconditioning” can result from a variety of environmental circumstances or situations. These include some that could not have been anticipated by evolution (i.e., space flight) but others are derived from the study of Earth bound circumstances (e.g., prolonged bedrest), and others not directly associated with the term “deconditioning” (e.g., a sedentary lifestyle such as sitting all day at a desk in front of a computer or TV). Some may become apparent during aging when loss of biologic regulatory control leads to compromised integrity and onset of diseases. Another example of this is menopause in females. Before menopause, females appear to be somewhat protected from cardiovascular events, but after menopause this advantage is apparently lost (reviewed in El Khoudary, 2020; Medin et al., 2020; Goossens et al., 2021). Similarly, after menopause subsets of females suffer from osteoporosis, osteoarthritis, dementia, and obesity at rates exceeding those of age-matched males. Thus, many risks for chronic diseases are “silent” prior to menopause, but evident after this biologic change. Therefore, some deconditioning environments are related to mechanical events, while others more related to biological transitions.

## The ‘Experiment” of Space Flight as a Model of Human “Deconditioning”

Over the past ~60 years, humans have engaged in space flight, with flights progressing from very short times to a year on the International Space Station (ISS) in Low Earth Orbit (LEO). Evolution could not have anticipated space flight and thus, the response of humans to space flight conditions with its associated microgravity. Therefore, studying humans in space can be viewed as an “experiment” to both identify in more detail how humans are regulated, and to identify and find solutions to enhance the health and safety of astronauts. Thus, from this “experiment,” it appears that human's exhibit heterogeneity in response to space conditions (reviewed in Scott et al., 2021), and that some of the same physiological systems affected by age/aging in many humans on Earth are also affected by space conditions (Ray, 1991; Vernikos and Schneider, 2010). As such, space flight may be viewed as “accelerated aging” (Ray, 1991; Vernikos and Schneider, 2010), or for the context of this discussion, acute (short term) and chronic (longer term) “deconditioning” based on removal from the boundary conditions of Earth and the mechanical activity required to maintain system integrity.

That this is so can be gleaned from the responses of astronauts to microgravity and removal of the 1 g variable, but still within the influence of the geomagnetic field of the Earth on the ISS. First, there is a rapid loss of muscle mass, some of which can be retained via the 2 h/day exercise program that some astronauts participate in as part of their daily regimen. This muscle loss is also accompanied by infiltration of fat in the muscle, further compromising function (discussed in Burkhart et al., 2019). Secondly, there is bone loss, but the loss is quite variable with some astronauts losing much less per month than others (range of 0.1–>1%). Interestingly, bone loss is only partially influenced by current exercise protocols (discussed in Ploutz-Synder et al., 2014; Sibonga et al., 2019), but apparently can be influenced by biophosphonates such as those that are prescribed for osteoporosis (LeBlanc et al., 2013), as on Earth. Thirdly, there are alterations to neuro systems related to control of vestibular functions (discussed in English et al., 2019), brain plasticity (Popova et al., 2020), and ocular alterations (Huang et al., 2019; Macias et al., 2020). In addition, there are alterations to the cardiovascular system which remain altered even in astronauts that perform the exercise routines. The changes to the cardiovascular system involve a redistribution of volume, alterations to carotid stiffness, and others (discussed in Hughson, 2009; Hughson and Shoemaker, 2015; Hughson et al., 2016, 2018). Thus, while exercise alone in space may be sufficient to prevent or reverse the effect of loss of gravity for muscle, other systems appear to require more than exercise to retain their integrity. Either such systems require chronic exposure to an artificial 1 g environment and the amount of exercise is not sufficient, or there are other boundary condition variables that are required to maintain their integrity. Interestingly, as the vast majority of astronauts have been males, some of the adaptations to space such as bone loss and cardiovascular alterations have yet to be thoroughly explored and characterized in females. However, as osteoporosis on Earth is mainly a disease of females (~75% female) (discussed in Karlamangla et al., 2018; RinonapoIi et al., 2021), the heterogeneous bone loss in males in space implies that there may be more than one regulatory system operative controlling bone integrity. Whether bone is unique in this respect remains to be determined.

Interestingly, the “reconditioning” of astronauts after return to Earth is also somewhat individual with respect to the rate of recovery, and whether recovery occurs (discussed in Ploutz-Synder et al., 2014). Also of interest is the report that some epigenetic changes occurring during space flight are reversible after the return to Earth, so at least some of the changes are apparently not permanent (Garrett-Bakelman et al., 2019). Therefore, heterogeneity exists for both the induction of deconditioning of biological systems during space flight (reviewed in Scott et al., 2021), as well as regarding the recovery, scenarios that could allow for investigation of the factors contributing to both facets of the deconditioning.

Thus, this cadre of astronauts would potentially be an excellent population to better understand human evolution regarding to how humans have adapted to living under the boundary conditions of Earth and the heterogeneity associated with such adaptations (Hart, 2018a). The population is fit, their health has been monitored continually for years while in the astronaut program, and their deconditioning responses to microgravity can be assessed. However, one drawback is the small number of individuals that actually go into space.

## Bedrest “Deconditioning” on Earth Parallels that of Space Flight

The “de-conditioning” observed in space environments can also be mimicked by prolonged bed rest, particularly with a 6-degree head down tilt, but also just with bed rest (reviewed in Hart and Zernicke, 2020). Bedrest differs from space flight in that in space, the gravity variable is removed, but in bedrest the 1 g variable is still present but the individual no longer works against it. However, there can be rapid muscle and bone loss, with the bone loss quite variable, similar to what has been observed in space (Kos et al., 2013), and cardiovascular deconditioning as well (Fortrat et al., 2001; Orter et al., 2020; Sandal et al., 2020; Solbiati et al., 2020), which again mimics the observations from space. In addition, chronic bedrest can lead to impairment of neuro-functions related to memory, and these can be reversed by exercise (Friedl-Werner et al., 2020). Some of the changes occurring after prolonged bedrest can persist for long after a return to normal activity (Trudel et al., 2009) and thus the system compromise from such deconditioning can be protracted even when reinstitution of physical activity is implemented. However, similar to space flight, the responses to bedrest can show considerable individual variation (Fernandez-Gonzalo et al., 2021; reviewed in Scott et al., 2021).

Of relevance is the fact that the deconditioning occurring with bedrest is that many aspects of it can occur quite rapidly even without the 6-degree head down tilt designed to mimic space flight deconditioning (Smorawinski et al., 2001; Kos et al., 2013) Whether such rapid deconditioning may be a function of age, and whether extensive conditioning blunts the rate and extent of deconditioning associated with bedrest is not known in any detail. It may also depend on the genetics of the individual given the heterogeneity of humans for a variety of physiological parameters and response patterns. Interestingly, fitness level prior to even a 3 day bedrest can influence the responsiveness to the bedrest and the type of exercise contributing to fitness level (e.g., endurance trained vs. strength trained) can influence the response pattern (Smorawinski et al., 2001).

The two examples discussed above indicate that evolution has placed conditions on how our essential systems function (i.e., mobility, navigation, cardiovascular, neuroregulatory) and their boundary conditions (i.e., 1 g, geomagnetic) affect integrity. However, while the two examples indicate that evolution has placed conditions on these systems, it does not address the optimization of the systems either individually, or as an integrated organism. Clearly, the individual systems are designed by evolutionary variables to function within a “physiological window” or “set point” (discussed in Turner, 1991; Hart and Scott, 2012), and there is considerable variation in the response to acute or chronic stimulation by an intervention such as exercise (aerobic or resistive patterned physical activity). There is also variation between individuals in responses to such exercise, as evidenced by the success and failure of athletes in different sports (discussed in Joulia-Ekaza and Cabello, 2006; Morrison and Cooper, 2006). There are also other examples, such, as representatives of groups from Kenya are excellent marathon runners (Onywere et al., 2006; Onywere, 2009; Tucker et al., 2015), while others are excellent at running short distances at high speed (likely an attribute good for escaping predators, as well as obtaining food sources) (Longman et al., 2015). Other populations have adapted to life at high altitude with lower oxygen levels via accommodations in respiratory and cardiovascular systems (Hanaoka et al., 2012; Forrer et al., 2021; Verrati et al., 2021). Thus, in part, optimal depends on the environmental conditions, and the ability to respond to challenges. In addition, for those in athletic endeavors, much of the variation that contributes to success might be silent under modern conditions, but has offered advantage during evolutionary processes.

The bedrest approach is potentially a very good avenue to explore the mechanisms involved in human deconditioning of several physiological systems. It can be well-controlled regarding the time involved (acute or chronic), it can involve individuals of varying ages, it allows for assessment of “before, during and after” scenarios, and it differs from that derived from astronauts in that it is performed in the presence of the 1 g environment. While the numbers per study are not high, since performed under controlled conditions, outcomes can be compared between studies. It should be noted that bedrest differs from space flight in that in bedrest on Earth, the 1 g environment can still likely influence some physiological systems while in space, the 1 g environment has been removed.

## Influence of a Sedentary Lifestyle on Deconditioning

While 24 h/day prolonged bedrest leads to deconditioning of multiple systems, a response that parallels responses to microgravity during spaceflight, a less intense but an equally serious form of “deconditioning” can occur with implementation of a sedentary lifestyle where the ratio of time/day spent in physical activity declines to the point where the risk for loss of system integrity becomes more pronounced (Thijssen et al., 2011; Nosova et al., 2014; Hughson and Shoemaker, 2015; Canu et al., 2019; Eanes, 2021; Perez-Lasierra et al., 2021; Stoner et al., 2021; Wilson et al., 2021). The development of this so called “sitting disease” has evolved in populations of both adults and younger individuals spending large amounts of their day sitting due to their occupation (e.g., sitting in front of a computer or at a desk all day), watching TV, or occupied with electronic devices rather than engaging in normal physical activities such as walking where ground reaction forces require work again the forces of gravity, or engaged in various forms of exercise (e.g., aerobic or resistive) (Dunstan et al., 2021; Hwang et al., 2021; Stoner et al., 2021). Thus, when activity levels fall below a threshold for maintenance of system integrity, the “use it or lose it” principle appears to become activated even though the inactivity is not constant as during 24 h/day bedrest. How such a threshold is established that must be broached by sedentary behavior on an individual basis is largely unknown but it may depend on genetics, activity levels during growth and maturation, and nutrition to name a few potential influences. However, the study of specific populations may provide clues to how it is maintained (Lazarus and Harridge, 2017).

While sedentary behavior is a risk factor for deconditioning in adults, it is also poses significant risks for those undergoing growth and development as the coordinate growth of bones, joints and muscles are likely dependent on physical activity and continued exposure to ground reaction forces (discussed in Hart, 2018a). Whether the effects of chronic sedentary behavior/deconditioning prior to skeletal maturity can be reversed as an adult remains to be determined.

Secondary to sedentary behavior is the risk for development of obesity with its own contributions for deconditioning (discussed below). This sedentary behavior plus obesity can have severe detrimental effects on the integrity of the MSK and cardiovascular systems for both adults and those undergoing growth and maturation, particularly as these conditions become chronic (Elagizi et al., 2020; Kanellopoulou et al., 2021). There is also some evidence for the shared interaction between these variables at the genetic level (Schnurr et al., 2021).

Risks associated with sedentary behavior can likely be mitigated by exercise (Tersa-Miralles et al., 2020; Farrahi et al., 2021; Gillen et al., 2021; Hatamoto et al., 2021; Peddie et al., 2021; Taylor et al., 2021), changing the behavior to breakup periods of sedentary behavior (Gouldrup and Ma, 2021), but certainly, as such behavior becomes more chronic and sustained, there is a risk for epigenetic alterations to the adaptations to inactivity that may pose risks for the reversibility and impact on conditions associated with aging and the impact of exercise (discussed in Barrons-Cabrera et al., 2019; Bagley et al., 2020; Rezus et al., 2020; Sellami et al., 2021). For many individuals, physical activity across the lifespan can likely impact the consequences of sedentary behavior and/or aging, possibly via the epigenetic influences of exercise or physical activity (Ling and Ronn, 2014).

Understanding the mechanisms associated with alterations induced by sedentary behavior is an active area of research (Pedersen and Febbraio, 2012; Guescini et al., 2015; Wu et al., 2015; Smits et al., 2018; Ghosh et al., 2019; Greco et al., 2019; Olanrewaju et al., 2020; Padilha et al., 2021; Hou et al., 2022). While such studies are providing new insights into the various considerations regarding sedentary behavior, the interpretation of the results of such studies may be complicated by the complexity of chronicity, human heterogeneity, age, sex, and other factors such as activity between bouts of hypo activity. However, the findings obtained from bedrest studies (a more extreme form of intermittent sedentary behavior) may also be subsequently applied to sedentary populations to better assess risk for associated complications and the effectiveness of interventions. Assessing deconditioning associated with bedrest and intermittent sedentary behavior could also lead to comparisons and identification of unique features of the genes and molecules involved in the two modalities (Smits et al., 2018), as well as compared to space flight responses where the 1 g environment variable has been removed.

## The “Deconditioning” Associated With Menopause

While not generally thought of as a “deconditioning” process, from some perspectives menopause may be defined as such for females. After the onset of puberty at ~11–12 years of age, females are exposed to cyclical variations in sex hormones for ~35+ years. During development of menopause or loss of ovarian function, a process usually covering several years after ~45 yoa, females lose ovarian production of sex hormones. Thus, after chronic exposure to such sex hormones for a protracted period of time, the production of most of the systemic sex hormones ceases, and there is an onset of a variety of conditions in subsets of females. These can include osteoporosis, increased risk for cardiovascular disease, dementia, osteoarthritis, obesity, and sarcopenia (discussed in Hussain et al., 2018; Khandelwal, 2020; Barnes and Charkoudian, 2021; Fischer and Haffner-Luntzer, 2021; Maas, 2021; Udeh-Momoh and Watermeyer, 2021). Not all females exhibit all of the conditions mentioned, but some can exhibit more than one. As the lifespan of humans has been extended dramatically over the past several decades in the developed world, the consequences of menopause may not have been a consideration for many females during evolution. Thus, the retention of the conditioning imposed by sex hormones likely serve functions regarding reproductive processes. Considerable change occurs during pregnancy, including vascular changes, bone adaptations, and immune/inflammatory responses (discussed in Brislane et al., 2021; Capozzi et al., 2021; Piccinni et al., 2021). For instance, in a rabbit ligament, it has been shown that neurovascular regulation is altered during pregnancy (McDougall et al., 1998, 2000).

Thus, after menopause, many systems, including those that are similar to the ones altered during space flight are influenced by menopause. Interestingly, exercise can apparently impact several of these systems, but not others. For example, exercise is reported to influence the progression of dementia in patients with the vascular type of disease (Davey, 2017; Barha and Liu-Ambrose, 2020; Schatz et al., 2020; Barha et al., 2021a,b) or cognition in older adults (Erickson and Kramer, 2009; Ma et al., 2016). While not as effective for inhibiting some changes associated with space flight, exercise and conditioning can contribute to retention of muscle and cardiovascular function on Earth.

While the deconditioning associated with menopause affects a large number of individuals, its study is complicated by the heterogeneous response to menopause. As this form of deconditioning is associated with a metabolic change (e.g., loss of hormonal regulation) rather than a consequence of physical inactivity, it does offer the potential to better understand the ability of constant reinforcement of conditioning on the loss of hormonal regulation. Integration of such findings with the outcomes of the space flight and bedrest studies could lead to significant advancement in the understanding of such relationships.

## “Deconditioning” Associated With Obesity

Currently, there is an “epidemic” of both adult and childhood onset obesity. Thus, in North America, the rate of adult obesity is ~35 and >70% are overweight, with the rates in children raising as well (discussed in Johnson and Johnson, 2015; Colmenarejo, 2020; Chrissini and Panagiotakos, 2021). In adult onset obesity, there is increased risk for cardiovascular disease (discussed in Budhram-Mahadeo et al., 2021; Nabrdalik et al., 2021), diabetes (reviewed in Cheng et al., 2021), dementia (discussed in Uddin et al., 2021), osteoarthritis (reviewed in Midgley, 2020; Sun et al., 2020), and loss of muscle integrity via infiltration with fat (reviewed in Collins et al., 2018). With over 100 genes linked to obesity risk (discussed in Froguel, 2015) and multi-generational epigenetic risk as well (discussed in Ahmed, 2010; Paul et al., 2019), realization of such risks likely may reside in sedentary behavior and poor nutrition associated with high fat high sugar diets of prepared foods (discussed in Collins et al., 2018). Thus, with adult onset obesity there is a “deconditioning” of the host with many consequences.

For many individuals with adult onset obesity, it is difficult to lose weight and those that do lose a large amount of weight, they have difficulty keep the weight off and often it returns (reviewed in Collins et al., 2018). Addition of an exercise program with a dietary program may not always affect weight loss, but it can lead to improved conditioning. Thus, the concept of “fit but fat” has evolved (discussed in Cheng et al., 2021), but it is not clear what the long term benefits are to this condition. However, having a conditioned musculoskeletal system and a conditioned cardiovascular system likely can lower the consequences of obesity.

The “deconditioning” occurring during childhood onset obesity may have some consequences in common with adult onset (i.e., metabolic syndrome, increased risks for cardiovascular disease, and type 2 diabetes), but may also have some unique consequences related to growth and maturation. Thus, “deconditioning” arising after birth but before puberty may influence the cardiovascular system, muscles, bone, joints and many other systems, and their coordination (i.e., systems biology from an integrated perspective) as they are growing and establishing a “set point” at skeletal maturity. Subsequently, the onset of puberty on such a “deconditioned” set of systems may be altered, and altered in a manner that could impact the potential for success or failure to re-establish conditioning later in life. Onset of obesity and “deconditioning” after puberty but before skeletal maturity may lead to an altered “physiological set point” for how systems coordinate, leading to impaired responses to conditioning after skeletal maturity, and for the females, and altered response to menopause. Whether such early “deconditioning” will lead to overt pathology that cannot be prevented or reversed by conditioning protocols after skeletal maturity, remains to be seen.

That some of the above discussion may have merit comes from preclinical models. In rats fed a high fat high sugar diet as adults, a significant subset develop joint damage (reviewed in Collins et al., 2018). However, if the animals are fed the same diet from the post weaning period for the same length of time, they do not develop joint damage (Collins et al., 2020). In adults, feeding that type of diet and subjecting the animals to either an exercise program and/or a prebiotic to impact the gut microbiome, prevents the consequences of the diet and associated obesity (Rios et al., 2019). Providing exercise and/or after joint damage had occurred reversed some aspects of the “deconditioning,” but not others (Rios et al., 2020). Whether such exercise regimens are effective during early life obesity induction remains to be investigated. However, it has been noted that in this rat model, exercise can prevent some of the impact of the diet and subsequent obesity on cardiac muscle (Boldt et al., 2021).

While the study of deconditioning associated with obesity induction and chronicity of obesity is complex and involves many physiological systems, there will likely be overlap between the different forms of deconditioning that allow for assessment of commonalities and unique features. For instance, >100 genes have been implicated in obesity (discussed in Hart, 2021), and while many may be unique to this form of deconditioning, some may overlap with other forms that have been discussed above.

## Acute vs. Chronic “Deconditioning”: An Issue that Needs to Be Addressed

An issue that goes beyond the characterization of “deconditioning” is the issue of acute vs. chronic deconditioning and the potential for reversing the effects of the “deconditioning.” First of all, humans are very heterogeneous as mentioned above, and therefore, the answer to this issue may be individual-specific, based on the nature of the deconditioning insult, or even be tissue or metabolic system-specific.

However, in other systems it is known that the acute insult may be reversible, while the chronic state may be more difficult to stop or reverse. Thus, acute inflammation can be impacted by anti-inflammatories, but the chronic inflammation associated with a condition such as rheumatoid arthritis may require different interventions, possibly due to epigenetic alterations (Nemtsova et al., 2019; Nygaard and Firestein, 2020). Similarly, acute pain and chronic pain may involve local and central mechanisms differently (discussed in Tucker-Bartley et al., 2021). In addition, by the time dementia has been diagnosed, it has been shown to be very difficult to stop progression. It is likely that the >100 genetic risk factors for “deconditioning” associated with obesity were perhaps associated with food insecurity during evolution and not the chronic state observed today. The conversion of the acute need for fat storage in times of food insecurity to a chronic state presently with its “deconditioning” may be why some people cannot control their weight even when a conditioning program is instituted subsequent to the development of chronic obesity.

Humans experience daily circadian rhythms, where there are periods of activity and inactivity (usually sleep) that are accompanied by variations in chemicals (i.e., corticosteroids, melatonin) (Majidinia et al., 2018) and controlled by neural loci such as the suprachiasmatic nucleus (SCN) and the hypothalamus (discussed in Hut and Van der Zee, 2011). Interestingly, such cycles are disrupted in young rats with obesity, similar to what is seen on old normal weight rats (discussed in Bravos Santos et al., 2016). While inactivity during sleep is normally considered restorative, from a mechanical loading perspective such acute periods of physical inactivity could be also considered an acute period of “deconditioning.” Thus, evolutionarily, such alternating periods of activity/inactivity were deemed important for system integrity and likely should be considered as part of the 24-h activity cycle (discussed in Rosenberger et al., 2019). With the disruption of these periods of activity/inactivity in the elderly (discussed in Bravos Santos et al., 2016), there may also be increased risk for the development of chronicity regarding disease development (Lazarus and Harridge, 2017). However, more robust periods of activity could lead to restoration of aspects of the circadian rhythms.

## Lessons Learned From the Study of “Conditioning/Deconditioning”

Thus, not only has the study of different “deconditioning” environments provided insights into the regulation of various physiologic systems related to the “use it or lose it” principle, they also have provided better understanding into what potential interventions are needed to re-establish conditioning. Of importance are potential differences between biomechanical variables such as ground reaction forces (GRF) in maintaining conditioning vs. metabolic factors such as sex hormones and obesity that can compromise the integrity of conditioning.

The study of “deconditioning” associated with space flight and prolonged bedrest, as well as sedentary behavior have also reinforced the concept that humans are quite heterogeneous in their responses to deconditioning environments, possibly indicating the complexity of understanding the genetic basis for such heterogeneity that may emerge during aging or as a consequence of bedrest on Earth for the elderly. This heterogeneity in responses, for example on the cardiovascular system likely cannot be explored in detail with astronauts due the small number of individuals that go into space presently, but could be explored in the future when space tourism becomes more of a reality. The numbers of individuals in bedrest studies is also not usually large due to cost, but studies that complement spaceflight associated studies in the future may provide valuable correlations. For bedrest studies, the length of time can vary from 3 days (Smorawinski et al., 2001) to 60 days (Basner et al., 2021) and there are usually 10–20 participants per study. Usually, the participants are often all males or females and young adults which means sex differences are not addressed within a specific study. However, relevant to this discussion, while in space for 1 year, epigenetic changes occurred in an astronaut, but not in his Earth-bound twin (Garrett-Bakelman et al., 2019). Interestingly, after returning to Earth, many of the epigenetic alterations in the astronaut twin had reverted to pre-flight levels. Thus, such approaches could be applied to larger numbers of individuals in the future to better understand variation in responses and epigenetic changes in specific genes and regulatory elements following exposure to ground-based deconditioning environments.

The metabolic deconditioning factors such as menopause and obesity are likely different from space flight and bedrest in that sex hormones and lipids, and the consequences of the obesity such as type II diabetes, metabolic syndrome and impact on vascular integrity are not biomechanically based and are more complicated to unravel their biologic complexity. The fact that only a subset of postmenopausal females become at risk for cardiovascular events or become obese means that the underlying sex-hormone influenced targets are uniquely regulated in that subset, a set of circumstances that could confound clinical trials. Similarly, factors related to obesity overlap with menopause and likely have links to the >100 postulated obesity related genetic risk factors (discussed in Froguel, 2015).

## Can We Use Some Forms of Deconditioning To Better Understand Risk For Diseases of the Affected Physiological Systems and Develop Specific Interventions?

As discussed above, there are multiple forms of “deconditioning” that can occur, some natural and others are somewhat artificial. Furthermore, the deconditioning associated with obesity and menopause are more challenging with regard to when they start [i.e., peri-menopausal state in females; shorter vs. longer term changes with an obesity-inducing diet (discussed in Collins et al., 2018)]. In contrast, the deconditioning associated with space flight and bedrest, as well as sedentary lifestyle studies, offer the unique ability to knowing when they start and stop, allowing for better control of the before and after environments.

While the number of astronauts is quite low, longer and farther space missions are planned and thus, having them subjected to bedrest deconditioning prior to space flight could allow for development of precision medicine approaches to address their responses to Earth-based deconditioning since there are parallels (discussed in Hart, 2018a). Certainly, there is a reluctance for astronauts to have their genomes sequenced if it could affect their subsequent time in space, but perhaps not all humans, given their heterogeneity, should actually be candidates for multi-year space flights which are very expensive. In addition, the responses in actual space flight (in the complete absence of gravity) may be different from bedrest or intermittent sedentary behavior changes (no GRF but gravity still present) so this would be important to ascertain.

Developing a cohort of young control males and females and assessing their biological responses (muscle, bone, cardiovascular, neurological) to a period of deconditioning bedrest, and then following them longitudinally for several decades could lead to insights into development of “diseases of aging” based on normally silent features of physiology. Assessing the genomes and gene expression phenotypes of the individuals in such cohorts may also elucidate genetic correlations with both responses to bedrest conditions and aging. Such an approach would again allow for assessment of risk going forward and development of effective interventions for people in Earth (including specific types of exercise and drugs, as well as lifestyle choices) who appear to lose integrity in similar systems during the aging process. Such insights could then be expanded to a more general population with genetic biomarkers for risk to expand the impact. There are likely some limitations to this approach, such as life-dependent epigenetic changes, but these changes could also be addressed via re-sequencing and potentially, reversing the impact of such changes. This approach could also be applied with the added variable of exercise to address genetic variation associated with cardiovascular disease (Hagberg, 2011).

Furthermore, such approaches as described above are long term considerations and should not be considered in isolation. Thus, the long term investigative approach should also be balanced by the more empirical approach of enhancing the efficacy of existing modalities that have proven to be successful, such as exercise protocols.

## Can We Improve On Aerobic and Resistance Exercise to Enhance Conditioning of Physiological Systems?

While aerobic and resistance loading of tissues and exposure to GRF are likely those biomechanical stimuli that were most associated with conditioning of physiological systems during the evolution of *Homo sapiens* to their current state, that process would likely be optimized only to the point where the physiologic systems enhanced survival to an average lifespan, and thus would not have been optimized to environmental conditions humans find themselves in currently. Certainly, athletic endeavors have revealed that there is considerable diversity on human potential to further optimize conditioning to excel at aerobic and/or resistive exercise associated with athletics. Whether these traits were selected for in specific tasks during evolution or were just stochastic events remain to be determined.

However, such loading activities were performed as part of everyday life during evolution and not restricted to specific events or times at a gym and designated exercises. Likewise, the “normal” exercise was not performed against a backdrop of a chronic condition such as prolonged bedrest or long duration space flight. Thus, to optimize the influence of exercises (either programed or normal daily exercise) one should likely develop a consistent pattern across the lifespan rather than to use “exercise” as a treatment for chronic deconditioning for most humans who are not bedridden or plan to venture into space for a prolonged time. Therefore, prevention of deconditioning or mitigating the risks for deconditioning of various physiological systems is a better solution to a potential problem. However, improvements could be made by personalizing exercise for the individual as some people respond better to resistance vs. aerobic exercises (discussed in Sparks, 2017; Hart and Zernicke, 2020), and it is known that different types of exercise can elicit differing responses (Baptista et al., 2008) including myokine patterns (Gonzalez-Gils and Elizonda-Montemayor, 2020; discussed in Hart and Zernicke, 2020). Certainly, in the case of sedentary behavior, frequent bouts of activity have been found to be better than a single bout of intense activity (Gouldrup and Ma, 2021). In contrast, regular bouts of intense exercise may be advantageous as a general approach to modify development of adverse molecular changes (Sellami et al., 2021). Thus, variation in physical activity and exercise with regard to duration, intensity and frequency may depend in part, on individual circumstances.

While a consistent pattern of activity may be the best solution to prevent deconditioning of systems such as the cardiovascular and musculoskeletal systems, as well as neuro-cognition systems, that is not to say that further improvements for some people cannot be made through augmentation with other interventions, particularly as humans' age (a factor that could be complicated by humans aging faster or slower than their chronological age). Therefore, some drugs could be used for those with particular genetic or stochastic compromise to allow for exercise/activity to exert a better outcome. However, we should likely not depend on the pharma approach alone to rectify a lack of varied exercises. Thus, to address the chronicity of some conditions to resist exercise-based interventions may require some pharma interventions to reverse epigenetic modifications contributing to the chronicity or better identification of hormone-dependent targets contributing to post-menopausal risk for cardiovascular conditions and obesity which could lead to targeted pharma interventions. Such interventions do not have to be complicated or expensive, as previously it was suggested that adding low doses of the non-patentable metabolic modulator lithium carbonate and/or prebiotics may enhance the benefits of exercise in relation to cognition (Hart, 2018b), but it may also be beneficial for other risk factors as well.

While the drug approach may be acceptable to some, the issue of nutrition is also paramount. A wide variety of “fad diets” have been touted over the years to address some of the deconditioning issues. Given human heterogeneity, it is likely that “one size does not fit all” regarding nutrition as well as exercise or physical activity. For many, it is not what you should eat, it is what you should avoid at different stages of the lifespan, likely including sugar and fat, prepared foods, and excess amounts of food (nutritional imbalance).

Finally, to enhance efforts in this area the development and validation of appropriate biomarkers to evaluate the impact of interventions on host systems would be beneficial, particularly with respect to exercise interventions. These could include some of the myokines that are released from muscle via exercises in both humans (Kim et al., 2015; Kelahmetoglu et al., 2020) and preclinical models (Zhang et al., 2017; Ahn and Kim, 2020). Of the myokines, irisin appears to be of significance at multiple levels and tissues including the heart (Seo et al., 2020), bone (Zhang et al., 2017; Bettis et al., 2018), and *in vitro* in space environments (Colucci et al., 2020). Thus, their study in several conditioning/deconditioning environments may be useful in the context indicated above.

Of relevance to the above discussion are findings related to heterogeneity in responsiveness to exercise programs in cohorts of humans across the lifespan (discussed in Garcia-Pinillos et al., 2016; Weatherwax et al., 2016; Alvarez et al., 2018; Saez de Asteasu et al., 2019). Thus, for many such programs, participants can be categorized as responders or non-responders. Attempts of overcome non-responder phenotype by altering the exercise programs is often stochastic and it is not clear why some participants do not respond (Hrubeniuk et al., 2021). This is an important category of individuals that need to be addressed in order to implement interventions to prevent or rescue from deconditioning. The study of deconditioning environments as discussed above may lead to genotypic and phenotypic characterization of individuals that associate with the responder and non-responder categories and therefore, allow for successful development of effective interventions to prevent deconditioning across the lifespan, but particularly during aging when deconditioning leads to apparent increased risk for disease development.

## Conclusions

From the above discussion, it is apparent that multi-system deconditioning is central to a number of biological circumstances, and this deconditioning interrupts normal biological integrity and it arises in part based on the evolutionary history contributing to *Homo sapiens* development (inactivity via bedrest or sedentary behavior, menopause, obesity, aging), while others could not have been anticipated by evolution (i.e., space flight). Thus, it is clear that mobility and associated exposure to GRF and aerobic/resistance-induced loading in a 1 g environment are essential for maintaining multiple biological systems system and deconditioning, or loss of integrity can result from separation from the 1 g environment (prolonged bedrest, space flight) or from metabolic alterations (menopause, obesity) or their consequences (i.e., diabetes and metabolic syndrome).

A critical element regarding the maintenance of conditioning is that humans are very heterogeneous, and thus, when it comes to exercise or patterned loading, “one size likely does not fit all” (Barha et al., 2021a,b) regarding those features of the above list. Of critical importance therefore, in any attempt to rectify this conundrum is to better define the minimum level and type of exercise/loading required to maintain the integrity of the various systems and the optimal level of exercise/loading that leads to prolonged integrity into aging. The variation between individuals due to their complement of evolutionary “idiosyncratic” elements that contribute to the definition of the “optimal” levels of exercise, and to better define the needs across of the lifespan of females and males (growth/maturation, post-puberty, skeletal maturity, post-menopause for the females, and in the >60 yoa population) will shape what is defined as optimal. Clearly, as a preventative, exercise is health and is central to who humans are, but in many of today's “modern” civilizations, what was part of everyday life has become a topic for specific events (i.e., participation in sport, going to the gym, programed exercise), or has disappeared from daily living.

As physical activity and physical conditioning has been ingrained in evolution and as such, evolution provided the tools to respond to exercise, that relationship has been hampered by modern civilizations, as well as abrogation of health to the health care system rather than retain it as an individual responsibility. Thus, the study of “deconditioning” environments support the need for humans to embrace the requirement for exercise/conditioning for maintaining system integrity, and not rely on medicine to fix the problems resulting from “deconditioning.” Improved understanding of the molecular basis associated with deconditioning in different environments will likely lead to enhanced precision health for individuals via tailored interventions, as well as how best to contravene such factors that may contribute to disease development during aging. Thus, such improved understanding of what happens when many physiological systems are not used above a threshold should have implications for both health and fundamental information on human heterogeneity at the level of systems developed under the boundary conditions of Earth.

## Author Contributions

The author confirms being the sole contributor of this work and has approved it for publication.

## Conflict of Interest

The author declares that the research was conducted in the absence of any commercial or financial relationships that could be construed as a potential conflict of interest.

## Publisher's Note

All claims expressed in this article are solely those of the authors and do not necessarily represent those of their affiliated organizations, or those of the publisher, the editors and the reviewers. Any product that may be evaluated in this article, or claim that may be made by its manufacturer, is not guaranteed or endorsed by the publisher.
